# The role of artificial intelligence in medical imaging research

**DOI:** 10.1259/bjro.20190031

**Published:** 2019-11-28

**Authors:** Xiaoli Tang

**Affiliations:** 1 Yale New Haven Hospital, New Haven, USA

## Abstract

Without doubt, artificial intelligence (AI) is the most discussed topic today in medical imaging research, both in diagnostic and therapeutic. For diagnostic imaging alone, the number of publications on AI has increased from about 100–150 per year in 2007–2008 to 1000–1100 per year in 2017–2018. Researchers have applied AI to automatically recognizing complex patterns in imaging data and providing quantitative assessments of radiographic characteristics. In radiation oncology, AI has been applied on different image modalities that are used at different stages of the treatment. *i.e*. tumor delineation and treatment assessment. Radiomics, the extraction of a large number of image features from radiation images with a high-throughput approach, is one of the most popular research topics today in medical imaging research. AI is the essential boosting power of processing massive number of medical images and therefore uncovers disease characteristics that fail to be appreciated by the naked eyes. The objectives of this paper are to review the history of AI in medical imaging research, the current role, the challenges need to be resolved before AI can be adopted widely in the clinic, and the potential future.

## A brief overview of the history

A handful of scientists from a variety of fields (mathematics, psychology, engineering, economics and political science) began to discuss the possibility of creating an artificial brain. They gathered together at a workshop held on the campus of Dartmouth College during the summer of 1956. This is widely known as Dartmouth Workshop, and it founded a society of artificial intelligence (AI).^1^ The field then went through its peaks and valleys several cycles. MIT cognitive scientist Marvin Minsky along with other attendees at the Dartmouth Workshop were extremely optimistic about AI’s future. They believed that AI will substantially be solved within a generation. However, no significant progress was made. After several criticizing reports and ongoing pressure from congress, government funding and interests dropped off. 1974–90 became the first AI winter. In the 80’s, due to the competition of the British and Japan, AI revived. 1983–93 was a major winter for AI, coinciding with the collapse of the market for the needed computer power, which led to withdrawal of funding again. Research began to pick up again after that. One well-known event was IBM’s Deep Blue—the first computer beat a chess champion. In 2011, the computer giant’s question answering system Watson won the quiz show Jeopardy, and this marked the newest wave of AI booming. In Parallel of recent 10 years in medical imaging research, the amount of imaging data has grown exponentially. This has increased the burden to physicians to process the images. They need to read images with higher efficiency while maintain the same or better accuracy. At the same time, fortunately, computational power has also grown exponentially. These challenges and opportunities have formed the perfect foundation for the AI to be blossomed in the medical imaging research.

Researchers have successfully applied AI in radiology to identify findings either detectable or not by the human eye. Radiology is now moving from a subjective perceptual skill to a more objective science.^2,3^ In Radiation Oncology, AI has been successfully applied to automatic tumor and organ segmentation,^4–6^ ^7^^8^ and tumor monitoring during the treatment for adaptive treatment. In 2012, a Dutch researcher, Lambin P, proposed the concept of “Radiomics” for the first time and defined it as follows: the extraction of a large number of image features from radiation images with a high-throughput approach.^9^ As AI became more popular and also more medical images than ever have been generated, these are good reason for radiomics to evolve rapidly. Radiomics is a novel approach for solving the issue of precision medicine. These researches have demonstrated a great potential of the role of AI in medical imaging. In fact, it has sparkled one of the ongoing discussions—will AI replace clinicians entirely? We believe it will not. For short term, AI is constrained by a lack of high quality, high volume, longitudinal, outcomes data, a constraint that is further exacerbated by the competing need for strict privacy protection.^10^ There were approaches to address the privacy threat, like distributed learning. However, in a 2017 paper, it was argued that any distributed, federated, or decentralized deep learning approach is susceptible to attacks that reveal information about participant information from the training set.^11^ For long term, we believe that AI will continue to underperform human level accuracy in medical decision making. Fundamentally, medicine is art, not science. AI might be able to outperform human in terms of quantitative tasks. Overall medical decision, however, will still depend on human evaluation to achieve the optimal results for a given patient.

## Current role of AI in radiology

Machine learning, as a subset of AI, also called the traditional AI, was applied on diagnostic imaging started 1980’s.^12^ Users first predefine explicit parameters and features of the imaging based on expert knowledge. For instance, the shapes, areas, histogram of image pixels of the regions-of-interest (*i.e.* tumor regions) can be extracted. Usually, for a given number of available data entries, part of them are used as training and the rest would be for testing. Certain machine learning algorithm is selected for the training to understand the features. Some examples of the algorithms are principal component analysis (PCA), support vector machines (SVM), convolutional neural networks (CNN), etc. Then, for a given testing image, the trained algorithm is supposed to recognize the features and classify the image.

One of the problems of machine learning is that users need to select the features which define the class of the image it belongs to. However, this might miss some contributing factors.^12,13^ For instance, lung tumor diagnosis requires user to segment the tumor region as structure features. Due to the patient and user variation, the consistency of the manual feature selection has always been a challenge. Deep learning, however, does not require explicit user input of the features. As its name suggests, deep learning learns from significantly more amount of data. It uses models of deep artificial neural networks. Deep learning uses multiple layers to progressively extract higher level features from raw image input. It helps to disentangle the abstractions and picks out the features that can improve performance. The concept of deep learning was proposed decades ago. Only till recent decade, the application of deep learning became feasible due to enormous number of medical images being produced and advancements in the development of hardware, like graphics processing units (GPU).^14^ However, with machine learning gaining its relevance and importance every day, even GPU became somewhat lacking. To combat this situation, Google developed an AI accelerator integrated circuit which would be used by its TensorFlow AI framework—tensor processing unit (TPU). TPU is designed specifically for neural network machine learning and would have potential to be applied on medical imaging research as well.

The main research area in diagnostic imaging is detection. Researchers started developing computer-aided detection (CAD) systems in the 1980s. Traditional machine learning algorithms were applied on image modalities like CT, MRI, and mammography. Despite a lot of effort made in the research area, the real clinical applications were not promising. Several large trials came to the conclusion that CAD has at best delivered no benefit^15^ and at worst has actually reduced radiology accuracy,^16^ resulting in higher recall and biopsy rates.^17,18^

The new era of AI—the deep learning has so far demonstrated promising improvements in the research area over the traditional machine learning. As an example, Ardila et al proposed a deep learning algorithm that uses a patient’s current and prior CT volumes to predict the risk of lung cancer.^19^ The model achieved a state-of-the-art performance (94.4% area under the curve) on 6716 national lung cancer screening trial cases and performed similarly on an independent clinical validation set of 1139 cases. As a comparison of conventional screening by low-dose CT, per cancer.gov,^20^ there are several associated harms: false-positive exams, overdiagnosis, complications of diagnostic evaluation, increase in lung cancer mortality, and radiation exposure. One false-positive exam example provided on the web site was 60%. Overdiagnosis was estimated at 67%. There is also radiation induced risk to develop lung cancer or other types of cancer later in life. AI-based diagnosis reduced these risks.

In fact, deep learning algorithms have become a methodology of choice for radiology imaging analysis.^20^ This includes different image modalities like CT, MRI, PET, ultrasonography etc and different tasks like tumor detection, segmentation, disease prediction etc. Researches have shown that AI/deep learning-based methods have substantial performance improvements over the conventional machine learning algorithms.^21^ Similar to human learning, deep learning learns from enormous amount of image examples. However, it might take much less time, as it solely depends on curated data and the corresponding metadata rather than the domain expertise, which usually takes years to develop.^12^ As the traditional AI requires predefined features and have shown plateauing performance over recent years, and with the current success of AI/deep learning in image research, it is expected that AI will further dominate the image research in radiology.

## Current role of AI in radiation oncology

In radiation oncology imaging research, AI has been applied in organ and lesion segmentation, image registration, fiducial/marker detection, radiomics etc. Similar to radiology, it started with traditional AI and now with deep learning.^3,22–24^^25^^26^ In the most recent *Medical Physics* journal (May 2019, Volume 46, Issue 5), there were 16/51 papers on deep learning-based imaging research. As we know, imaging research is only one subsection of the entire radiation oncology research. The large portion of the published deep learning imaging research articles demonstrates the important role AI is now playing in the field.

For organ and lesion segmentation, the main goal is to segment the organs at risk automatically for treatment planning. Deep learning algorithms have been applied to segment head and neck organs, brain, lung, prostate, kidney, pelvis etc. Lesion segmentation applications include bladder, breast, bone, brain, head and neck, liver, lung, lymph nodes, rectum etc. Sahiner et al^23^ has summarized the segmentation object, deep learning methods used, data set used, and the corresponding performance. One algorithm used often was U-net.^27^ Unlike traditional AI, U-nets consist of several convolution layers, followed by deconvolution layers, with connections between the opposing convolution and deconvolution layers. The network can therefore analyze the entire image during training and allow for obtaining segmentation likelihood maps directly.

Dong et al applied U-net-generative adversarial network (U-Net-GAN) to train deep neural networks for the segmentation of multiple organs on thoracic CT images.^28^ U-Net-GAN jointly trains a set of U-Nets as generators and fully convolutional networks (FCNs) as discriminators. The generator and discriminator compete against each other in an adversarial learning process to produce the optimal segmentation map of multiple organs. The proposed algorithm was demonstrated feasible and reliable in segmenting five different organs. Similarly, Feng et al successfully applied deep convolutional neural networks (DCNN) for thoracic organs at risks segmentation using cropped hree-dimensional images.^29^ CNN has also been used on head and neck organ segmentation.^30^

Holistically nested networks (HNN) uses side outputs of the convolutional layers, and it has been applied on prostate and brain tumor segmentation.^31,32^

In radiation therapy, often there are needs to register one image modality to another (multimodal) or an image on a one day to another (monomodal). To avoid traditional AI which required handcrafted features, an unsupervised deep learning feature selection framework was proposed. It implemented a convolutional stacked auto-encoder network to identify the intrinsic features in image patches.^33^ The algorithm demonstrated better Dice ratio scores compares to state of the art. These can be applied on both multimodal and monomodal image registrations. Sloan et al^34^ have proposed a novel method of image registration by regressing the transformation parameters using a convolutional neural network (CNN). This was applied on both mono- and multimodal applications. With the promising result AI has demonstrated so far in the research domain, we hope the AI-based image registration can be applied in the clinic soon. This is an important step towards real-time adaptive treatment planning and delivery.

The automatic fiducial/marker detection is needed for real time tracking of the treatment area during the delivery. Most common methods require prior knowledge of the marker properties to construct a template. Recent proposed deep learning CNN framework requires no prior knowledge of marker properties or additional learning periods to segment cylindrical and arbitrarily shaped fiducial markers.^22^ The algorithm achieved high classification performance.

Radiomics, one of the most advanced AI applications in medical imaging research, is a novel approach towards the precision medicine.^35^ Radiomics consists two steps. First step is feature extraction. Images from multiple modalities might be included. Image segmentation algorithms are applied to segment the volumes of interest. After the segmentation, features will be extracted. Common features include texture, geometric information, tumor volume, shape, density, pixel intensity etc. The second step is to incorporate the extracted features into mathematical models to decoding the phenotype of the tumor for treatment outcome prediction. A successful outcome prediction can provide valuable information for precise treatment design. For instance, different lung cancer patients might share many similarities like histology and age. However, the images of the tumor might appear different, and the survival time might be very different.^36^ If radiomics can take the image information, decode the phenotype, and therefore predict the survival time or prognosis prior to the treatment, different treatment regimens might be chosen. This is called personalized or precision medicine.^35^ Traditionally, precision medicine depended on biomarkers to estimate patient different prognosis or subtype, which usually required invasive biopsy. Radiomics, on the other hand, does not require invasive procedures. It was shown that features extracted from CT images of lung cancer patients alone correlate well with gene mutations and have prognostic powers.^37^ The success of radiomics can potentially avoid undesirable complications caused by biopsy^38,39^ and achieve the same or better prediction outcome.

Aerts et al^37^ built a radiomic signature, assessed on an independent lung data set. It demonstrated the translational capability of radiomics across different cancers. Authors also showed significant associations between the radiomic features and gene-expression patterns. Some researchers did radiomics modeling using positron emission tomography (PET) images,^40^ PET/CT or PET/MRI.^41^ Most applications were on lung cancer. There are also applications on head and neck^42^ and prostate cancers.^43^ All these models have achieved reasonable prediction power.

### Challenges need to be resolved before clinical implementation

Despite the excitement AI has generated in the medical imaging research, there are challenges before it can become more robust and be widely adopted in the clinic. AI is constrained by a lack of high quality, high volume, longitudinal, outcomes data. Even the same image modality on the same disease site, the parameters of the imaging setting and protocols might be different in different clinical settings. Each set of images is associated with a clinical scenario. The number of potential clinical scenarios and the variety of tasks that each of the image might contain is astronomical and might be impossible to be tacked by one organization with any AI algorithm. Each patient cohort associated with a clinic is different. The way each clinic practices is also different. How to organize the data generated from different practices in a more standard way is a big challenge on AI-based medical imaging research. Medical imaging data organization itself might deserve to be a major research field.

There are challenges associated with medical imaging data curation.^44^^45^ Data curation is an important step. Accurate labeling therefore is a key. As the exponentially growth of the number of images, clinicians have challenges to process them with the same efficiency and accuracy. It usually takes years to train people to become experts. Therefore, the lack of ability to keep up labeling enormous number of images imposes limitations of the data curation.

On the policy level, there are increasing concerns on patient privacy. Patient-related health information was protected by tight privacy policies, which limited cross-institution image sharing. Recently, there were several headline news level healthcare data breaches and security attacks. As a result, hospitals are now more than ever concerning about securities and liabilities and have tightened up security and data sharing policies. However, the success implementations of AI needs large amount of data from multiple institutions. How to share images without compromising security is a challenge.

## The future of AI in medical imaging research

Two challenges need to be resolved before AI can be more widely implemented in medical imaging research. First, how to organize and pre-process data generated from different institutions. Miotto et al stated in their breakthrough work “deep patient”—challenges in summarizing and representing patient data prevent widespread practice of predictive modeling using electronic health records. They presented a novel unsupervised deep feature learning method to derive a general-purpose patient representation from electronic health record data that facilitates clinical predictive modeling.^46^ Authors have successfully derived patient representations from a large-scale data set that were not optimized for any specific task and can fit different clinical applications. However, their data are from one institution. Tackling data set from multiple institutions in fact is a much more challenging task. Even for the same procedure, different institution might implement differently. Patient cohorts might also be different. All these will need to be addressed when pre-process data for AI algorithm.

Second, on a policy or infrastructure level, how to encourage more image data sharing is also a challenge. Currently, image data sharing is very limited. HIPAA compliant is one concern, and lack of infrastructure is another. The medical data security needs to work with the emerging needs of data sharing. Corresponding infrastructure also needs to be built.

On the long run, how AI can become true “intelligent” at the human level is a key to the question if AI can replace human in medical imaging. Unlike pure quantitative task, the knowledge involved in medical imaging related decision making require life experience and philosophy. For the machine to behave in human level, there are not only challenges on data collection and algorithm development, but also on ethical regulations.

## Conclusions

AI is playing a significant role in medical imaging researches. It changed the way people process the enormous number of images. There are still challenges to be resolved before AI can eventually impact clinical practices.
